# A Comparative Study of Optimizing Genomic Prediction Accuracy in Commercial Pigs

**DOI:** 10.3390/ani15070966

**Published:** 2025-03-27

**Authors:** Xiaojian Chen, Yiyi Liu, Yuling Zhang, Zhanwei Zhuang, Jinyan Huang, Menghao Luan, Xiang Zhao, Linsong Dong, Jian Ye, Ming Yang, Enqin Zheng, Gengyuan Cai, Jie Yang, Zhenfang Wu, Langqing Liu

**Affiliations:** 1 National Engineering Research Center for Breeding Swine Industry, College of Animal Science, South China Agricultural University, Guangzhou 510642, China; a2742144723@gmail.com (X.C.); yiyiliu0921@outlook.com (Y.L.); yulingzhang@stu.scau.edu.cn (Y.Z.); zwzhuang@outlook.com (Z.Z.); huangjinyan.scau@hotmail.com (J.H.); lmh968974@163.com (M.L.); zhaox133@163.com (X.Z.); eqzheng@scau.edu.cn (E.Z.); cgy0415@163.com (G.C.); jieyang2012@hotmail.com (J.Y.); wzfemail@163.com (Z.W.); 2 Guangdong Zhongxin Breeding Technology Co., Ltd., Guangzhou 510642, China; linsong325@163.com (L.D.); jye1992@126.com (J.Y.); 3 Guangdong Provincial Key Laboratory of Agro-Animal Genomics and Molecular Breeding, Guangzhou 510642, China; 4 College of Animal Science and Technology, Zhongkai University of Agriculture and Engineering, Guangzhou 510225, China; yangming@zhku.edu.cn

**Keywords:** ssGBLUP, marker density, genomic prediction accuracy, cross-validation, carcass and body traits

## Abstract

Genomic prediction (GP), which uses genome-wide markers to estimate breeding values, is a crucial tool for accelerating genetic progress in livestock and plant breeding. The accuracy of GP depends on several factors, including the statistical model, marker density, and cross-validation strategy. This study evaluated these factors to optimize GP accuracy for eight economically important carcass and body traits in a Duroc × (Landrace × Yorkshire) (DLY) pig population. This study used 50 K SNP chip data from 1494 DLY pigs, which were imputed to the whole genome sequence (WGS) level. Seven different models were compared, including GBLUP, ssGBLUP, and five Bayesian models. The ssGBLUP model consistently outperformed other models across all traits, with prediction accuracies ranging from 0.371 to 0.502. Further analyses showed that prediction accuracy improved with increasing cross-validation folds and marker density, particularly in the low-density panel. However, the improvement plateaued in medium-to-high-density scenarios. These findings underscore the importance of carefully selecting the model, marker density, and cross-validation strategy to optimize GP accuracy for carcass and body traits in commercial pigs. The insights from this study can guide breeders and researchers in maximizing genetic progress in pig breeding programs.

## Simple Summary

Genomic prediction (GP) is a revolutionary technique using DNA markers to predict an animal’s genetic potential, aiding breeders in making informed selection choices. This study explored factors affecting GP accuracy for eight economically important carcass and body traits in commercial pigs. These traits, including body length, height, backfat thickness, and loin muscle area, impact pig production efficiency and profitability. This research assessed seven different prediction models and found that the ssGBLUP model, which integrates both pedigree and genomic data, consistently provided the most accurate predictions. This model outperformed other commonly used models, including GBLUP and various Bayesian approaches. This study also highlighted the importance of marker density. Increasing the number of genetic markers used, particularly in low-density panels, led to improved prediction accuracy. Finally, using a larger number of cross-validation folds during model evaluation enhanced prediction accuracy. This research emphasizes that carefully selecting the appropriate model, marker density, and cross-validation strategy is crucial for optimizing GP in pig breeding programs and maximizing genetic progress for desirable traits.

## 1. Introduction

Genomic prediction (GP), a revolutionary technique first proposed by Meuwissen et al. [1], has transformed animal and plant breeding by enabling the estimation of breeding values using genome-wide markers. This method, based on the concept that at least one marker is in linkage disequilibrium (LD) with a quantitative trait locus (QTL) that affects the trait of interest, allows breeders to predict genomic estimated breeding values (GEBVs) for individuals in a candidate population. This is achieved by constructing a model using phenotype and genotype data from a training population, facilitating the early selection of individuals with desirable traits, improving overall population performance, and significantly reducing the costs associated with traditional breeding practices [2,3].

As genotyping costs decrease, GP is being widely adopted in animal and plant breeding programs. However, the accuracy of GP is not uniform across all traits and is significantly influenced by factors such as the statistical model used, the number of cross-validation folds, and the density of markers used in the analysis. Different statistical models employed in GP can be broadly categorized into direct and indirect methods. Direct methods, such as Genomic Best Linear Unbiased Prediction (GBLUP), utilize kinship matrices derived from genomic information to directly estimate breeding values [4]. In contrast, single-step GBLUP (ssGBLUP), a more sophisticated approach, combines both genomic and pedigree data to further enhance prediction accuracy [5]. Indirect methods, such as ridge regression BLUP (RRBLUP) and Bayesian methods, first estimate the effects of individual markers and then use these estimates to calculate individual GEBVs [6,7].

Each statistical model comes with its own set of assumptions and may be more suitable for certain traits depending on their underlying genetic architecture. For instance, Bayesian models are often considered to be more biologically realistic as they allow for different prior distributions for marker effects. Additionally, semi-parametric approaches like Reproducing Kernel Hilbert Space (RKHS) and non-parametric machine learning methods are also used for genomic prediction [8,9,10]. However, the choice of the most appropriate model depends on the specific trait under consideration and the genetic structure of the population. The number of cross-validation folds used in model evaluation also plays a critical role in determining the accuracy of GP. Kristensen et al. [11] reported that using a larger number of folds leads to improved prediction accuracy compared to using fewer folds, such as in a two-fold cross-validation scheme. Similarly, marker density, which refers to the number of markers used in the analysis, is another key factor influencing GP accuracy. Studies have demonstrated that increasing marker density, particularly in low-density panels, results in enhanced accuracy as it increases the likelihood of capturing causal mutations [12].

Here, we focus on optimizing GP for eight economically important carcass and body measurement traits in a commercial Duroc × (Landrace × Yorkshire) (DLY) pig population. We utilize a comprehensive approach to evaluate seven different GP models, including GBLUP, ssGBLUP, and five Bayesian models, to determine the model that provides the highest prediction accuracy for the selected traits. Furthermore, this study will assess the impact of different cross-validation folds, ranging from 2 to 10, on prediction accuracy. Lastly, the influence of marker density and variant type on prediction accuracy will be investigated using imputed whole genome sequencing (WGS) data, where variant type is categorized into single-nucleotide polymorphisms (SNPs) as well as insertions and deletions (INDELs). By addressing these key factors, this study aims to offer valuable insights into optimizing GP accuracy for carcass and body measurement traits, providing practical guidance for breeders and researchers aiming to maximize genetic progress in pig breeding programs.

## 2. Materials and Methods

### 2.1. Ethics Statement

All animals used in this study met the guidelines for the care and use of experimental animals established by the Ministry of Agriculture of China. The whole study was performed with the approval of the ethics committee of South China Agricultural University (Guangzhou, China) under SCAU#2014-0136.

### 2.2. Experimental Animals and Phenotypic Data

The experimental animals were a three-way crossbred commercial DLY pig population. A total of 1494 DLY pigs born between 2018 and 2019 were provided by Guangdong Wens Foodstuff Group Co., Ltd. (Yunfu, Guangdong, China). All pigs were raised under the same conditions. Phenotypic records included body length (BL), body height (BH), chest circumference (CC), waist circumference (WC), abdominal circumference (AC), loin muscle area (LMA), loin muscle depth (LMD), and backfat thickness (BF). In addition to the original phenotype, we included sex, farm, year–month of birth, and slaughter lot as fixed effects, using blupf90 software (PREDICTF90 ver. 1.7) [13] to correct phenotypes. The corrected phenotypes (y_c_) were used for subsequent analyses.

### 2.3. Genotyping and Imputation

The genomic DNA was extracted from ear tissue using standard phenol/chloroform methods [14], and the quality of DNA was determined using electrophoresis and the ratios of light absorption (A_260/280_ and A_260/230_). DNA samples were diluted to 50 ng/μL for genotyping procedures as described by Ding et al. [15]. The 1494 DLY pigs were genotyped with the GeneSeek Porcine 50 K Chip (Neogen, Lincoln, NE, USA), and quality control was conducted using PLINK software (v1.90) [16]. The criteria were as follows: individual call rate > 90%, SNP call rate > 90%, and minor allele frequency (MAF) > 5%. Moreover, only SNPs situated on the autosome chromosomes were retained. After quality control, 45,687 SNPs were retained for further analysis. Then, we imputed genotypes from 50 K to the whole genome sequence (WGS) level using SWIM [17]. SWIM is a pig haplotype reference panel, which was developed based on 2259 whole genome sequenced animals representing 44 pig breeds and demonstrated robust performance in genotype imputation, achieving a concordance rate above 96% and an r2 of 0.85. Then, we divided the imputed WGS data into SNP, INDEL, and SNP + INDEL datasets. The “--thin-count” parameter of PLINK software was used to further partition them into different marker densities of 1 K, 3 K, 7 K, 10 K, 30 K, 100 K, 500 K, and 1000 K. In this study, we treated 1 K, 3 K, and 7 K as low-density, 10 K, 30 K, and 100 K as medium-density, and 500 K and 1000 K as high-density data sets.

### 2.4. Genetic Parameter Estimation

Heritability was defined as the ratio of the additive genetic variance to phenotypic variance, and the formula is $\frac{\sigma_{g}^{2}}{\;\sigma_{g}^{2}+\sigma_{e}^{2}}$. Variance components were estimated using the restricted maximum likelihood (REML) algorithm via the GCTA 1.93.2 software [18].

### 2.5. Statistical Models

Seven different models were used for genomic prediction of carcass and body measurement traits in DLY pigs, including GBLUP, ssGBLUP, BayesA, BayesB, BayesC, Bayesian LASSO, and BayesR models.

#### 2.5.1. GBLUP

The GBLUP model is as follows:

y=μ+Zg+e,

where *y* is a vector of the corrected phenotypes, *μ* is the overall mean, g is the vector of additive genetic values, following a normal distribution of g~*N* (0, *G*$\sigma_{g}^{2}$), where $\sigma_{g}^{2}$ is the additive genetic variance, and *G* is the marker-based genomic relationship matrix. *Z* is the design matrix for g, *e* is the vector of random residual effect, following a normal distribution of *e*~*N* (0, *I*$\sigma_{e}^{2}$), where $\sigma_{e}^{2}$ is the residual variance, and *I* is the identity matrix. The equations of the mixed model are as follows:

$$\left[\begin{array}{cc} X^{\prime }X\; & X^{\prime }Z\\ Z^{\prime }X & Z^{\prime }Z+kG^{-1} \end{array}\right]\;\left[\begin{array}{c} \hat{b}\\ \hat{g} \end{array}\right]=\left[\begin{array}{c} X^{\prime }y\\ Z^{\prime }y \end{array}\right],$$

where $k=\sigma_{e}^{2}/\sigma_{g}^{2}$; the *G* matrix was constructed using the method proposed by VanRaden [19]:

$$G=\frac{MM^{\prime }}{2\sum_{i=1}^{m}p_{i}\left(1-p_{i}\right)},$$

where M is the genotype matrix, *m* is the number of markers, and *p_i_* is the minor allele frequency of the *i*th marker.

#### 2.5.2. ssGBLUP

ssGBLUP has the same model as GBLUP, and the corresponding mixed model equations are as follows:

$$\left[\begin{array}{cc} X^{\prime }X\; & X^{\prime }Z\\ Z^{\prime }X & Z^{\prime }Z+kH^{-1} \end{array}\right]\;\left[\begin{array}{c} \hat{b}\\ \hat{g} \end{array}\right]=\left[\begin{array}{c} X^{\prime }y\\ Z^{\prime }y \end{array}\right],$$

where *G* was substituted by *H*, the combined genotype and pedigree relationship matrix, and the *H*^−1^ matrix [20,21] was constructed using the following formula:

$$H^{-1}=A^{-1}+\left[\begin{array}{cc} 0 & 0\\ 0 & \left(G^{-1}-A_{22}^{-1}\right) \end{array}\right],$$

where *A* is the pedigree-based relationship matrix; *A*_22_ is the pedigree-derived relationship matrix for genotyped individuals; *G* is the genomic relationship matrix.

#### 2.5.3. Bayesian Models

The BLUP-based genomic prediction model assumes that all markers have the same genetic variance, while in practice only a few SNPs have an effect. The Bayesian models assume that the marker effect follows some prior distribution. These models require the estimation of marker effects in a reference population in which all individuals were phenotyped and genotyped, and the accumulation of marker effects in combination with the genotype information of the candidate population are used to obtain the individual GEBV [22]. All Bayesian models in this study were computed using the BGLR package [23]. The total number of iterations (nIter) for each cross-validation run was set to 12,000, with the burnIn parameter set to 2000, meaning that the first 2000 iterations were discarded to allow the chain to reach a stable state. The remaining settings were set to the default values provided by the BGLR package. The following model can be used to estimate the marker effects:

$$y=\mu +\sum_{i=1}^{m}X_{i}g_{i}+e,$$

where *y*, *μ*, and *e* are the same as in GBLUP, $g_{i}$ is the effect of the ith marker, *m* is the total number of markers, and *X* is the design matrix for g. The three genotypes AA, AB, and BB were represented by 0, 1, and 2, respectively. Five Bayesian models were used to estimate breeding values in this study: BayesA, BayesB, BayesC, Bayesian LASSO, and BayesR. The primary difference between these models is the assumption of the distribution of marker effects. In BayesA, it is assumed that all markers have effects that follow the normal distribution $g_{i}$~*N* (0, $\sigma_{g_{i}}^{2}$). The variances of the marker effects $\sigma_{g}^{2}$ are assumed to be distributed as an inverse chi-square distribution. The method uses Gibbs sampling into Markov chain Monte Carlo theory (MCMC) to calculate marker effects. BayesB assumes that a few markers have an effect, and most have zero effects, with an arbitrarily selected probability Π. And for those nonzero effects, the variances of marker effect follow the inverse chi-square distribution. When Π = 0, it is similar to BayesA. In BayesC, Π follows a uniform distribution Π~U (0, 1). Bayesian LASSO [24] assumes that the exponential distribution is the prior distribution of SNP effects. In BayesR [25], it is assumed that SNPs either have zero effect, a very small effect, a small effect, or a moderate effect. SNP effects are sampled from one of four possible normal distributions: *N*~(0, 0 ∗ $\sigma_{g}^{2}$), *N*~(0, 0.0001 ∗ $\sigma_{g}^{2}$), *N*~(0, 0.001 ∗ $\sigma_{g}^{2}$), and *N*~(0, 0.01 ∗ $\sigma_{g}^{2}$). These models calculate GEBV as follows:

$$GEBV=\sum_{i=1}^{m}X_{i}g_{i}$$

### 2.6. Genomic Prediction Accuracy and Bias

Genomic prediction accuracy was assessed using a cross-validation procedure. N-fold cross-validation means that all individuals were randomly and equally divided into *n* groups. One of these groups was treated as the validation set and the *n* − 1 group was treated as the training set. The cycle is conducted n times to ensure that each group can be a validation set once. We used N = 5 for accuracy assessment. The correlation between the GEBV and their corrected phenotypes in the validation set was used to calculate the accuracy of the genomic prediction [26]. The regression slope of *y_c_* on GEBV was used to measure the bias of the prediction, where a regression coefficient of 1 indicates no bias. We reported average prediction accuracy and bias values for 25 cross-validation rounds per trait.

## 3. Results

### 3.1. Statistical Phenotypes and Estimation of Heritability

This study examined the phenotypic variation and estimated the heritability of eight important traits in a commercial Duroc × (Landrace × Yorkshire) (DLY) pig population. These traits encompass five body measurements (BL, BH, CC, WC, and AC) and three carcass traits (LMA, LMD, and BF) (Table 1). These traits play a significant role in pig production and are key determinants of economic value. The coefficients of variation, which provide a measure of the relative spread of the data, ranged from 5.63% to 27.07%. LMA had the highest coefficient of variation, indicating the greatest variability, while BH had the lowest, suggesting less variability among individuals for this trait. The estimated heritability for the eight traits ranged from 0.39 to 0.52, signifying a medium level of heritability. BL had the highest heritability estimate, indicating a stronger genetic influence on this trait, while BF had the lowest.

### 3.2. Prediction Accuracy of Seven Statistical Models

**Figure 1:**
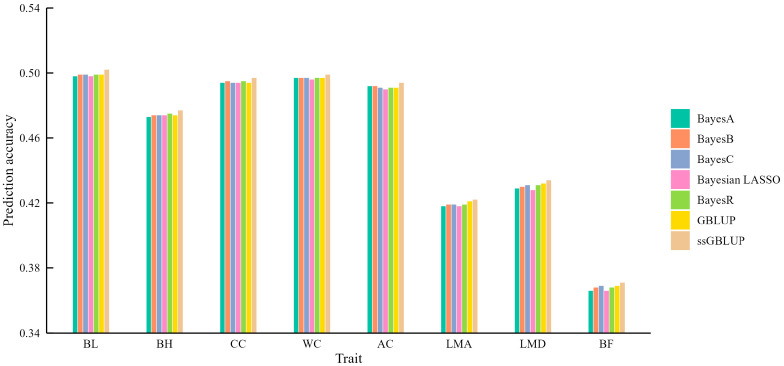
The mean accuracy of genomic prediction using seven different models for carcass and body measurement traits.

We herein used 50 K chip data and a five-fold cross-validation strategy for genomic prediction. ssGBLUP consistently outperformed the other models, achieving prediction accuracies ranging from 0.371 for BF to 0.502 for BL. Compared to GBLUP, the highest improvement was 0.003 for BL, BH, CC, and AC, while the lowest increase was 0.001 for LMA and LMD (Table S1). While the improvements over the other models were relatively small, this finding aligns with previous research demonstrating the superiority of ssGBLUP, attributed to its integration of both genomic and pedigree information, unlike GBLUP, which relies solely on genomic data (Figure 1).

The five Bayesian models, which incorporate different assumptions about the distribution of marker effects, did not consistently outperform GBLUP. For instance, none of the Bayesian models surpassed GBLUP or ssGBLUP for LMA and LMD, suggesting that the prior distribution assumptions might not be well-suited for these traits. For BF, only BayesC matched the accuracy of GBLUP but remained inferior to ssGBLUP. These findings underscore the notion that no single model is universally superior, and that model selection should be tailored to the specific trait being analyzed.

### 3.3. Prediction Accuracy of Different Cross-Validation Folds

**Figure 2:**
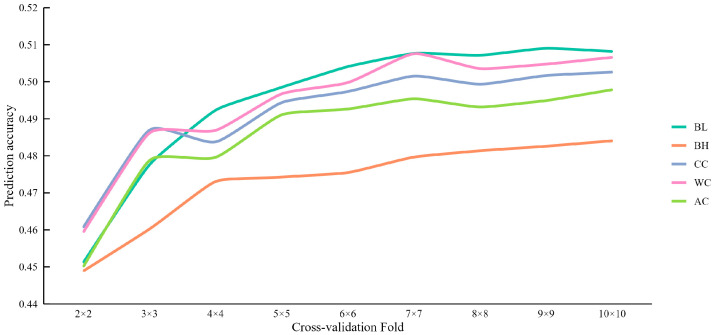
The mean accuracy of genomic prediction using different cross-validation folds for carcass and body measurement traits.

To explore the effect of the cross-validation folds on the accuracy of genomic prediction, we conducted genomic predictions for the five body measurement traits in the DLY population using nine different fold levels, ranging from 2 to 10 (Table S2). The results clearly demonstrated that increasing the number of cross-validation folds generally led to an improvement in prediction accuracy, which eventually stabilized at higher fold numbers (Figure 2). Seven-fold cross-validation achieved the highest prediction accuracy for WC. The most significant gains in prediction accuracy were observed when increasing the number of folds from 2 to 3.

### 3.4. Estimated Heritability of SNPs and INDELs from WGS

To investigate how marker density affects prediction accuracy, we categorized imputed WGS data into three variant types, including SNPs, INDELs, and a combination of SNPs + INDELs. These datasets were further divided into different marker densities, ranging from 1 K to 1000 K. The estimated heritability of the body measurement and carcass traits captured by SNPs and INDELs with different marker densities are inferred (Table S3). We found no significant difference in estimated heritability between SNPs and INDELs at low, medium, and high density. However, as marker density increased, the estimated heritability also increased. Using the estimated heritability from the 1 K marker dataset as a reference point, substantial increases were observed when the marker density reached the 1000 K level. The estimated heritability increased by 6% to 193% for SNPs, 25% to 86% for INDELs, and 3% to 150% for the combined SNPs + INDELs.

### 3.5. Prediction Accuracy of SNPs and INDELs from WGS

**Figure 3:**
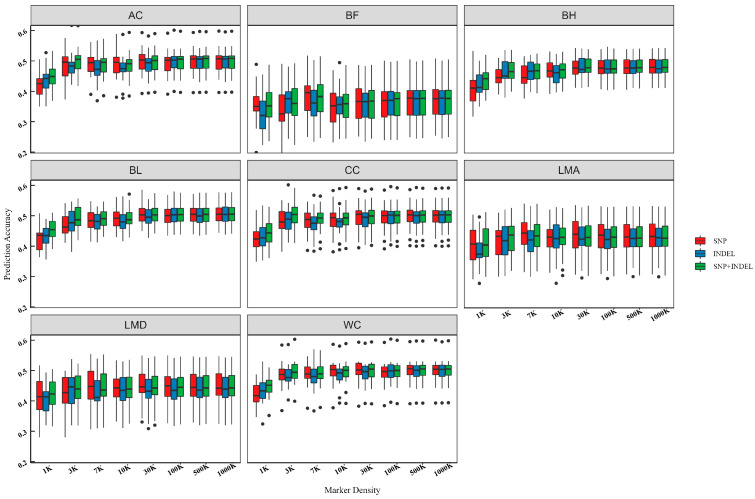
Box plot of genomic prediction accuracy over marker density for carcass and body measurement traits. “SNP” refers to the accuracy of genomic prediction using SNP. “INDEL” refers to the accuracy of genomic prediction using INDEL. “SNP + INDEL” refers to the accuracy of genomic prediction using SNP and INDEL.

We used the GBLUP model for genome prediction based on different variant types and densities and evaluated its prediction accuracy and bias with five-fold cross-validation. The results are shown in Table S4. A clear trend of increasing prediction accuracy with increasing marker density was observed, particularly within the low-density panels (Figure 3). Our analysis also revealed that in specific cases, the prediction accuracy of low-density INDELs exceeded that of low-density SNPs. For example, the prediction accuracy for BH was 3.6% higher with the 3 K INDELs compared to the 3 K SNPs. This finding suggests that INDELs can be valuable markers for genomic prediction, particularly in low-density panels. Using the 1 K marker dataset as a baseline, our results demonstrated substantial improvements in prediction accuracy for BL and AC when utilizing the 1000 K marker datasets. The 1000 K SNPs improved BL prediction accuracy by 19.1%, while the 1000 K INDELs improved it by 16.4%. Similarly, for AC, the 1000 K SNPs and INDELs led to improvements of 18.5% and 13.3%, respectively. Notably, the combined SNPs + INDELs dataset consistently demonstrated high prediction accuracy across different marker densities. Interestingly, we also found that SNPs at 30 K often outperformed both INDELs and SNPs + INDELs at the same density.

## 4. Discussion

### 4.1. Model Performance and Trait Architecture

In this study, we used 50 K chip data to perform genomic predictions for eight body measurement and carcass traits in DLY population. Two direct methods (GBLUP and ssGBLUP) and five indirect methods (BayesA, BayesB, BayesC, Bayesian LASSO, and BayesR) were utilized. Prediction accuracies varied across models and were trait-dependent. However, ssGBLUP consistently demonstrated the highest prediction accuracies, ranging from 0.371 to 0.502. The superior performance of ssGBLUP is attributed to its integration of both genomic and pedigree information, leading to a more comprehensive representation of genetic relationships compared to GBLUP, which relies solely on genomic data [27,28,29]. This combined approach is particularly advantageous in commercial settings, where pedigree data may be incomplete or unavailable.

The choice of the most effective genomic prediction model appears to be influenced by the underlying genetic architecture of the trait. For traits potentially governed by a large number of genes with small effects, GBLUP and ssGBLUP performed comparably to or better than Bayesian models [30]. This was observed for loin muscle area (LMA) and loin muscle depth (LMD), where none of the Bayesian models outperformed GBLUP or ssGBLUP. Daetwyler et al. [31] concluded that Bayesian models perform similarly or slightly worse than GBLUP when traits are affected by numerous small-effect QTL but perform better when influenced by only a few major QTL. In such cases, the more robust and computationally efficient GBLUP and ssGBLUP models might be preferred. While Bayesian models are often favored for traits that are significantly influenced by a few major genes, no single Bayesian model consistently outperformed the others across all traits in this study. The core of the Bayesian model lies in the setting of prior parameters. The genetic background of different traits is different, leading to different effect distributions of SNP loci affecting traits. This highlights the challenge of selecting the optimal Bayesian model for specific traits with unknown genetic architectures.

### 4.2. Factors Affecting the Accuracy of Genomic Prediction

Beyond model selection, several factors impact genomic prediction accuracy. The heritability of a trait plays a crucial role, as higher heritability generally translates to more accurate predictions. The estimated heritability for body measurement and carcass traits ranged from 0.39 (BF) to 0.52 (BL). Do et al. [32] reported moderate-to-high heritability for body measurement traits, consistent with our findings, though their eye muscle area exhibited a lower value of 0.296. Zhou et al. [33] estimated heritability of 0.34 and 0.25 for BL, 0.05 and 0.25 for BH, 0.30 and 0.27 for CC in Chinese Laiwu pigs at 210 and 240 days of age. Similarly, Song et al. [34] reported heritability of 0.5 for BL and 0.3 for BH. However, the heritability values of LMA and BF in our study were lower than those reported by Lo et al. [35], who found the value for LMA to be 0.80 and BF to be 0.56. The heritability of traits significantly impacts prediction accuracy. For instance, the BL trait, which had the highest heritability in our study, also had the most accurate predictions. Clevenland et al. [36] found a positive correlation between trait heritability and prediction accuracy. Traits with low heritability require a larger number of phenotype records for accurate marker effect estimation, as noted by de Roos et al. [37].

The number of cross-validation folds employed also affects prediction accuracy. Increasing the number of folds generally leads to improved accuracy, with the accuracy stabilizing as the number of folds increases further. In this study, prediction accuracy improved with an increase in cross-validation folds, with ten-fold cross-validation providing the highest accuracy. A substantial gain in accuracy was observed from two-fold to three-fold cross-validation settings for all traits, suggesting that even a small increase in folds can considerably enhance prediction accuracy.

Marker density also influences prediction accuracy. As expected, prediction accuracy improved with increasing marker density, especially in low-density datasets [37,38]. Higher marker densities encompass more causal mutations, reducing the gap between markers and causal mutations [39]. However, this improvement plateaus in medium-to-high-density scenarios, suggesting that beyond a certain point, adding more markers does not necessarily translate to better predictions. For instance, van Binsbergen et al. [40] found no advantage in using imputed WGS data over BovineHD genotype data in Holstein Friesian cattle, and Zhang et al. [41] reported little benefit from increasing marker density for predicting backfat depth and average daily feed intake. Once prediction accuracy plateaus, additional markers become redundant. Thus, a moderate marker density is often adequate to ensure each QTL is linked to at least one marker, leading to better predictions. This finding is important for practical breeding applications, as it suggests that a moderate marker density might suffice to achieve satisfactory prediction accuracy.

### 4.3. Implications for Pig Breeding

This study provides valuable insights into optimizing genomic prediction strategies for body measurement and carcass traits in DLY pigs. The findings, particularly the superior performance of ssGBLUP and the impact of marker density and cross-validation folds, can guide breeders in making more informed decisions regarding model selection and data utilization to maximize prediction accuracy and genetic progress. However, it is important to acknowledge that this study’s findings are specific to the DLY population and the traits examined. Future research should investigate the generalizability of these findings to other pig breeds and traits to further refine genomic prediction strategies for pig breeding.

## 5. Conclusions

In this study, we compared various statistical models and found that ssGBLUP performed better than both GBLUP and Bayesian models. ssGBLUP consistently outperformed GBLUP and Bayesian models, emphasizing the value of integrating genomic and pedigree data. Increasing the number of cross-validation folds led to improved prediction accuracy. Marker density also influenced prediction accuracy, with gains observed as density increased, particularly in low-density datasets. This improvement plateaued at medium-to-high densities, suggesting that a moderate density may suffice for accurate predictions. These findings provide a framework for optimizing genomic selection to enhance genetic progress in DLY pig breeding.

## Figures and Tables

**Table 1:** Statistical phenotypes and heritability of carcass and body measurement traits in DLY pigs.

Trait	N ^9^	Mean (±SD) ^10^	Min ^11^	Max ^12^	C.V./% ^13^	h^2^ (±SE) ^14^
BL (cm) ^1^	1494	123.70 ± 7.14	101.00	145.00	5.77	0.52 ± 0.02
BH (cm) ^2^	1494	64.60 ± 3.64	51.00	78.00	5.63	0.49 ± 0.02
CC (cm) ^3^	1494	112.24 ± 8.16	88.00	140.00	7.27	0.48 ± 0.02
WC (cm) ^4^	1494	110.45 ± 8.79	84.00	140.00	7.96	0.49 ± 0.02
AC (cm) ^5^	1494	121.34 ± 8.59	94.00	150.00	7.08	0.48 ± 0.02
BF(mm) ^6^	650	11.40 ± 3.08	5.50	20.80	27.07	0.39 ± 0.02
LMA(cm^2^) ^7^	650	40.36 ± 7.29	20.25	59.10	18.05	0.42 ± 0.02
LMD (mm) ^8^	650	53.18 ± 6.69	33.23	69.10	12.56	0.45 ± 0.02

^1^ Body length (BL). ^2^ Body height (BH). ^3^ Chest circumference (CC). ^4^ Waist circumference (WC). ^5^ Abdominal circumference (AC). ^6^ Backfat thickness (BF). ^7^ Loin muscle area (LMA). ^8^ Loin muscle depth (LMD). ^9^ Number of phenotype records (N). ^10^ Standard deviations (SD). ^11^ Minimum (Min). ^12^ Maximum (Max). ^13^ Coefficient of variation (C.V.). ^14^ Heritability (standard error) value (h^2^ (±SE)).

## Data Availability

The data that support the findings of this study are available on request from the corresponding author upon reasonable request. The data are not publicly available due to privacy or legal restrictions, because they belong to a commercial breeding company.

## Supplementary Materials

The following supporting information can be downloaded at https://www.mdpi.com/article/10.3390/ani15070966/s1: Table S1: The mean accuracy and standard error of genomic prediction from a five-fold cross-validation using seven models; Table S2: The mean accuracy and standard error of genomic prediction using different cross-validation folds; Table S3: The estimated heritability of different marker density SNPs and INDELs from WGS (h^2^ ± SE); Table S4: The mean accuracy and bias of genomic prediction using different densities of SNPs and INDELs (Mean ± bias).

## Abbreviations

The following abbreviations are used in this manuscript:

AC	Abdominal circumference
BF	Backfat thickness
BH	Body height
BL	Body length
BLUP	Best Linear Unbiased Prediction
CC	Chest circumference
GWAS	Genome-wide association study
GBLUP	Genomic Best Linear Unbiased Prediction
GEBV	Genomic estimated breeding value
GP	Genomic prediction
h^2^	Heritability
INDEL	Insertion and deletion
LD	Linkage disequilibrium
LMA	Loin muscle area
LMD	Loin muscle depth
MAF	Minor allele frequency
MCMC	Markov chain Monte Carlo theory
QTL	Quantitative trait loci
RRBLUP	Ridge Regression Best Linear Unbiased Prediction
SNP	Single-nucleotide polymorphism
ssGBLUP	Single-step Genomic Best Linear Unbiased Prediction
LASSO	Least absolute shrinkage and selection operator
WC	Waist circumference
WGS	Whole genome sequence
