# Supplementary material for: A Comparative Study of Optimizing Genomic Prediction Accuracy in Commercial Pigs
Source: Animals (Basel). 2025 Mar 27;15(7):966. doi: 10.3390/ani15070966 (PMC11988176; doi:10.3390/ani15070966)
Supplement: Supplementary file 1 [file animals-15-00966-s001.zip › Table S1.pdf]

Table S1. The mean accuracy and standard error of genomic prediction from a five-fold cross-validation using seven models

Model	BL	BH	CC	WC	AC	LMA	LMD	BF
GBLUP	0.499 (0.002)	0.474 (0.001)	0.494 (0.001)	0.497 (0.001)	0.491 (0.003)	0.421 (0.006)	0.432 (0.005)	0.369 (0.004)
ssGBLUP	0.502 (0.002)	0.477 (0.001)	0.497 (0.001)	0.499 (0.001)	0.494 (0.003)	0.422 (0.006)	0.434 (0.005)	0.371 (0.004)
BayesA	0.498 (0.002)	0.473 (0.001)	0.494 (0.001)	0.497 (0.001)	0.492 (0.003)	0.418 (0.006)	0.429 (0.005)	0.366 (0.003)
BayesB	0.499 (0.002)	0.474 (0.001)	0.495 (0.001)	0.497 (0.001)	0.492 (0.003)	0.419 (0.006)	0.430 (0.005)	0.368 (0.004)
BayesC	0.499 (0.002)	0.474 (0.001)	0.494 (0.001)	0.497 (0.001)	0.491 (0.003)	0.419 (0.006)	0.431 (0.005)	0.369 (0.004)
Bayesian LASSO	0.498 (0.002)	0.474 (0.001)	0.494 (0.001)	0.496 (0.001)	0.490 (0.003)	0.418 (0.006)	0.428 (0.004)	0.366 (0.004)
BayesR	0.499 (0.002)	0.475 (0.001)	0.495 (0.001)	0.497 (0.001)	0.491 (0.003)	0.419 (0.006)	0.431 (0.005)	0.368 (0.004)
