# Supplementary material for: A Comparative Study of Optimizing Genomic Prediction Accuracy in Commercial Pigs
Source: Animals (Basel). 2025 Mar 27;15(7):966. doi: 10.3390/ani15070966 (PMC11988176; doi:10.3390/ani15070966)
Supplement: Supplementary file 1 [file animals-15-00966-s001.zip › Table S2.pdf]

Table S2. The mean accuracy and standard error of genomic prediction using different cross-validation folds

Fold number	BL	BH	CC	WC	AC
2×2	0.451 (0.001)	0.449 (0.003)	0.461 (0.004)	0.459 (0.001)	0.450 (0.002)
3×3	0.478 (0.004)	0.460 (0.003)	0.487 (0.002)	0.486 (0.002)	0.479 (0.002)
4×4	0.492 (0.002)	0.473 (0.002)	0.484 (0.001)	0.487 (0.001)	0.480 (0.003)
5×5	0.499 (0.002)	0.474 (0.001)	0.494 (0.001)	0.497 (0.001)	0.491 (0.003)
6×6	0.504 (0.001)	0.475 (0.001)	0.497 (0.001)	0.500 (0.001)	0.493 (0.001)
7×7	0.508 (0.001)	0.480 (0.001)	0.502 (0.001)	0.508 (0.001)	0.495 (0.001)
8×8	0.507 (0.001)	0.481 (0.001)	0.499 (0.002)	0.504 (0.001)	0.493 (0.001)
9×9	0.509 (0.001)	0.483 (0.001)	0.502 (0.001)	0.505 (0.001)	0.495 (0.001)
10×10	0.508 (0.001)	0.484 (0.000)	0.503 (0.001)	0.507 (0.000)	0.498 (0.001)
