# Supplementary material for: A Comparative Study of Optimizing Genomic Prediction Accuracy in Commercial Pigs
Source: Animals (Basel). 2025 Mar 27;15(7):966. doi: 10.3390/ani15070966 (PMC11988176; doi:10.3390/ani15070966)
Supplement: Supplementary file 1 [file animals-15-00966-s001.zip › Table S3.pdf]

Table S3. The estimated heritability of different marker density SNPs and INDELs from WGS (h^2 ± SE)

Marker density	BL			BH			CC			WC		
	SNP	INDEL	SNP+ INDEL	SNP	INDEL	SNP+ INDEL	SNP	INDEL	SNP+ INDEL	SNP	INDEL	SNP+ INDEL
1K	0.29±0.03	0.29±0.03	0.36±0.04	0.27±0.03	0.30±0.03	0.34±0.04	0.27±0.03	0.29±0.03	0.33±0.04	0.27±0.03	0.28±0.03	0.33±0.04
3K	0.40±0.04	0.42±0.04	0.48±0.04	0.36±0.04	0.40±0.04	0.44±0.04	0.40±0.04	0.40±0.04	0.45±0.04	0.41±0.04	0.40±0.04	0.45±0.04
7K	0.45±0.04	0.44±0.04	0.48±0.04	0.40±0.04	0.44±0.04	0.45±0.04	0.45±0.04	0.41±0.04	0.46±0.04	0.44±0.04	0.41±0.04	0.45±0.04
10K	0.48±0.04	0.46±0.04	0.49±0.04	0.44±0.04	0.42±0.04	0.45±0.04	0.47±0.04	0.43±0.04	0.47±0.04	0.48±0.04	0.44±0.04	0.48±0.04
30K	0.55±0.04	0.51±0.04	0.54±0.04	0.50±0.04	0.49±0.04	0.51±0.04	0.52±0.04	0.48±0.04	0.51±0.04	0.52±0.04	0.47±0.04	0.50±0.04
100K	0.53±0.04	0.53±0.04	0.54±0.05	0.50±0.05	0.50±0.05	0.50±0.05	0.49±0.05	0.50±0.05	0.50±0.05	0.48±0.04	0.50±0.04	0.50±0.05
500K	0.55±0.04	0.53±0.04	0.54±0.04	0.51±0.05	0.50±0.05	0.51±0.05	0.51±0.05	0.50±0.05	0.50±0.05	0.50±0.05	0.49±0.05	0.50±0.05
1000K	0.55±0.04	0.54±0.04	0.55±0.04	0.51±0.05	0.50±0.05	0.51±0.05	0.51±0.05	0.50±0.05	0.51±0.05	0.51±0.05	0.50±0.05	0.50±0.05
	AC			LMA			LMD			BF		
	SNP	INDEL	SNP+ INDEL	SNP	INDEL	SNP+ INDEL	SNP	INDEL	SNP+ INDEL	SNP	INDEL	SNP+ INDEL
1K	0.27±0.03	0.30±0.03	0.34±0.04	0.34±0.06	0.28±0.06	0.35±0.06	0.35±0.06	0.31±0.06	0.38±0.07	0.35±0.06	0.28±0.06	0.36±0.07
3K	0.42±0.04	0.41±0.04	0.47±0.04	0.37±0.07	0.37±0.07	0.40±0.07	0.38±0.07	0.40±0.07	0.42±0.07	0.33±0.07	0.35±0.07	0.37±0.07
7K	0.46±0.04	0.41±0.04	0.46±0.04	0.44±0.07	0.41±0.07	0.45±0.08	0.45±0.07	0.41±0.07	0.46±0.07	0.42±0.07	0.37±0.07	0.42±0.08
10K	0.47±0.04	0.42±0.04	0.47±0.04	0.43±0.07	0.41±0.07	0.44±0.08	0.46±0.07	0.42±0.07	0.46±0.08	0.37±0.07	0.36±0.07	0.38±0.07
30K	0.52±0.04	0.47±0.04	0.50±0.04	0.47±0.08	0.44±0.07	0.46±0.08	0.49±0.07	0.45±0.07	0.47±0.08	0.40±0.08	0.37±0.08	0.39±0.08
100K	0.49±0.05	0.50±0.05	0.50±0.05	0.45±0.08	0.43±0.08	0.44±0.08	0.47±0.08	0.44±0.08	0.46±0.08	0.40±0.08	0.39±0.08	0.39±0.08
500K	0.51±0.05	0.50±0.05	0.51±0.05	0.46±0.08	0.44±0.08	0.45±0.08	0.48±0.08	0.46±0.08	0.47±0.08	0.40±0.08	0.40±0.08	0.40±0.08
1000K	0.51±0.05	0.50±0.05	0.51±0.05	0.45±0.08	0.44±0.08	0.45±0.08	0.48±0.08	0.46±0.08	0.47±0.08	0.41±0.08	0.40±0.08	0.41±0.08
