# Supplementary material for: A Comparative Study of Optimizing Genomic Prediction Accuracy in Commercial Pigs
Source: Animals (Basel). 2025 Mar 27;15(7):966. doi: 10.3390/ani15070966 (PMC11988176; doi:10.3390/ani15070966)
Supplement: Supplementary file 1 [file animals-15-00966-s001.zip › Table S4.pdf]

Table S4. The mean accuracy and bias of genomic prediction using different densities of SNPs and INDELs (Mean ± bias)

Trait	Variants type	Marker density							
		1K	3K	7K	10K	30K	100K	500K	1000K
BL	SNP	0.425±1.003	0.467±0.995	0.484±1.003	0.488±1.004	0.504±1.007	0.501±1.006	0.504±1.006	0.506±1.007
	INDEL	0.432±1.011	0.478±1.010	0.480±1.009	0.481±1.001	0.494±1.004	0.502±1.004	0.500±1.005	0.503±1.005
	SNP+INDEL	0.453±1.006	0.491±1.003	0.491±1.007	0.491±1.002	0.502±1.006	0.502±1.005	0.503±1.006	0.505±1.006
BH	SNP	0.407±1.011	0.446±1.009	0.451±1.004	0.466±1.004	0.476±1.000	0.478±1.001	0.479±1.000	0.482±1.002
	INDEL	0.420±1.014	0.462±1.001	0.468±1.005	0.461±1.005	0.478±1.000	0.476±0.998	0.479±1.001	0.480±1.001
	SNP+INDEL	0.438±1.014	0.469±1.003	0.467±1.005	0.468±1.004	0.479±1.000	0.478±1.000	0.480±1.001	0.481±1.002
CC	SNP	0.420±0.999	0.482±1.008	0.485±1.005	0.490±1.012	0.497±1.012	0.493±1.011	0.498±1.011	0.498±1.011
	INDEL	0.431±1.015	0.486±1.021	0.475±1.014	0.479±1.010	0.490±1.012	0.494±1.010	0.494±1.010	0.496±1.011
	SNP+INDEL	0.445±1.007	0.498±1.016	0.487±1.010	0.489±1.011	0.496±1.012	0.494±1.011	0.497±1.011	0.497±1.011
WC	SNP	0.419±1.003	0.485±1.002	0.487±1.007	0.497±1.005	0.501±1.007	0.495±1.008	0.500±1.010	0.501±1.010
	INDEL	0.434±1.018	0.483±1.014	0.480±1.017	0.485±1.006	0.491±1.011	0.499±1.010	0.498±1.010	0.499±1.010
	SNP+INDEL	0.447±1.012	0.497±1.008	0.490±1.012	0.495±1.006	0.498±1.009	0.498±1.009	0.500±1.010	0.500±1.010
AC	SNP	0.419±1.008	0.483±1.007	0.491±1.001	0.491±1.007	0.497±1.005	0.492±1.005	0.496±1.006	0.496±1.006
	INDEL	0.437±1.013	0.482±1.011	0.471±1.007	0.475±1.005	0.487±1.009	0.494±1.007	0.495±1.006	0.495±1.006
	SNP+INDEL	0.450±1.012	0.497±1.008	0.488±1.004	0.487±1.006	0.494±1.007	0.494±1.006	0.496±1.006	0.496±1.006
LMA	SNP	0.400±1.045	0.413±1.043	0.440±1.052	0.426±1.036	0.436±1.056	0.432±1.062	0.432±1.058	0.432±1.057
	INDEL	0.386±1.058	0.420±1.074	0.418±1.044	0.426±1.061	0.426±1.060	0.423±1.060	0.427±1.058	0.427±1.058
	SNP+INDEL	0.407±1.052	0.425±1.058	0.433±1.049	0.429±1.049	0.432±1.058	0.428±1.061	0.430±1.058	0.430±1.057
LMD	SNP	0.408±1.031	0.424±1.038	0.446±1.040	0.439±1.025	0.448±1.046	0.441±1.050	0.442±1.045	0.442±1.045
	INDEL	0.405±1.045	0.431±1.060	0.422±1.040	0.431±1.048	0.431±1.048	0.431±1.048	0.434±1.045	0.434±1.045
	SNP+INDEL	0.422±1.038	0.435±1.049	0.439±1.041	0.438±1.037	0.441±1.047	0.436±1.049	0.439±1.045	0.439±1.045
BF	SNP	0.358±1.020	0.340±1.025	0.384±1.044	0.352±1.043	0.367±1.051	0.364±1.059	0.368±1.053	0.372±1.054
	INDEL	0.322±1.030	0.364±1.072	0.357±1.050	0.357±1.057	0.356±1.060	0.362±1.054	0.366±1.058	0.367±1.057
	SNP+INDEL	0.356±1.035	0.362±1.050	0.376±1.046	0.357±1.050	0.363±1.056	0.364±1.057	0.368±1.056	0.369±1.056
